# New Insights into Key Determinants for Adenosine 1 Receptor Antagonists Selectivity Using Supervised Molecular Dynamics Simulations

**DOI:** 10.3390/biom10050732

**Published:** 2020-05-07

**Authors:** Giovanni Bolcato, Maicol Bissaro, Giuseppe Deganutti, Mattia Sturlese, Stefano Moro

**Affiliations:** 1 Molecular Modeling Section (MMS), Department of Pharmaceutical and Pharmacological Sciences, University of Padova, via Marzolo 5, 35131 Padova, Italy; giovanni.bolcato.1@studenti.unipd.it (G.B.); maicol.bissaro@studenti.unipd.it (M.B.); mattia.sturlese@unipd.it (M.S.); 2 School of Life Sciences, University of Essex, Wivenhoe Park, Colchester CO4 3SQ, UK; gd17863@essex.ac.uk

**Keywords:** GPCRs, adenosine receptors, selectivity, A_1_AR, A_2A_AR, antagonist, moleculardynamics (MD), supervised molecular dynamics (SuMD)

## Abstract

Adenosine receptors (ARs), like many otherGprotein-coupledreceptors (GPCRs), are targets of primary interest indrug design. However, one of the main limits for the development of drugs for this class of GPCRs is the complex selectivity profile usually displayed by ligands. Numerous efforts have been madefor clarifying the selectivity of ARs, leading to the development of many ligand-based models. The structure of the AR subtype A_1_ (A_1_AR) has been recently solved, providing important structural insights. In the present work, we rationalized the selectivity profile of two selective A_1_AR and A_2A_AR antagonists, investigating their recognition trajectories obtained by Supervised Molecular Dynamics from an unbound state and monitoring the role of the water molecules in the binding site.

## 1. Introduction

Adenosine receptors (ARs) are class A G protein-coupled receptors (GPCRs) that bind the endogenous agonist adenosine.ARs are composed of foursubtypes: A_1_, A_2A_, A_2B_, A_3_. While A_1_ and A_3_ARs (which share 49% of a sequence identity) are preferentially coupled to Gα_i_proteinsand therefore inhibit the adenylatecyclase, A_2A_ and A_2B_ARs (sharing 59% of a sequence identity) stimulatethis enzyme, as being coupled to Gα_s_ proteins [1]. Several ARs antagonists are in clinical trials for various diseases. With regards to A_2A_AR, is tradefylline has been recently approved for Parkinson’sdisease (NCT02610231) [2], PBF-509 is in phase I/II trials for non-small cell lung cancer (NCT02403193), and CPI-144 is in phase I trials for various cancer types (NTC02655822). PBF-680, on the other hand, is the only A_1_ AR antagonist inclinical phase II, for the treatment of Asthma (NTC02635945) [3].

One of the difficulties during the development of ARs agonists and antagonists as therapeutic agents is the poor selectivity between differentreceptorssubtypes [4]. For this reason, many efforts have been madeto elucidate the molecular basis of ARs ligands selectivity, and several structure-activity relationship (SAR) models have been developed for selective ligandsof all the four subtypes [1,2,5,6,7,8,9,10,11,12,13,14].

**Figure 1:**
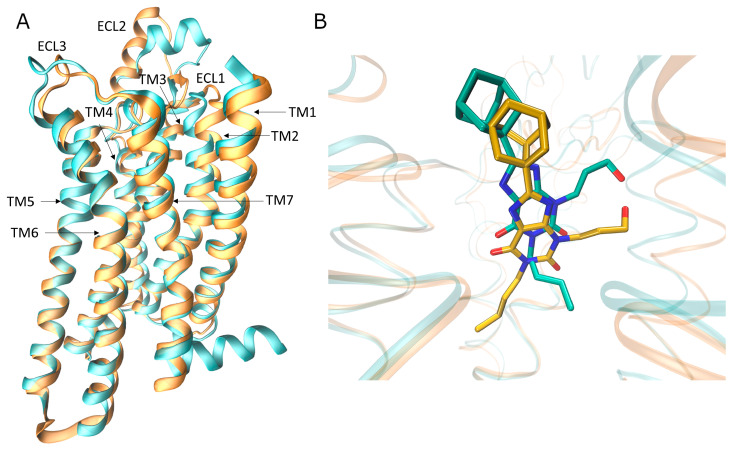
Comparison of A_1_ARand A_2A_AR structures (**A**) Superposition of the crystal structure of the inactive A_1_ (orange) and A_2A_ (cyan) adenosine receptors (Ars) (PDB code 5N2S and 5N2R respectively). (**B**) Superposition of the same xanthine ligand PSB36 in the two aforementioned crystal structures.

With the increasing availability of structural information (mainly from mutagenesis, X-ray, and cryo-EM approaches [15]) in the last few years, light has been shed on the origin of selectivity onARs.Recently, the A_1_AR inactive (PDB, Protein Data Bank, code 5UEN [16] and 5N2S [17]) and active (PDB code 6D9H [18]) structures have been solved. Interestingly, in [17], Cooke and colleagues obtained the X-ray crystal structure of A_1_ and A_2A_ Ars, in a complex with the same xanthine ligand PSB36, providing insight about the selectivity. There are several structural differences between A_1_AR and A_2A_AR. The second extracellular loop (ECL2), in particular, is more folded in A_1_AR and orients perpendicularly to the plane of the membrane, while in A_2A_ ARit forms a longer helix, which is parallel to the lipid bilayer. This difference is probably due to the presence of two disulfide bondsuniquely present in A_2_AR. Indeed, the bond between Cys71 and Cys159 anchorsECL2 to ECL1,while the bond between Cys74 and Cys146 tethers TM3 to ECL2. The class A conserved disulfide bonds between Cys80 and Cys169 is present in both the two subtypes. These differences in the disulfide bonds likely contribute to the outward movement of the top of transmembrane helix 2 (TM) in the inactive A_1_AR (Figure 1).

Further divergence involves TM7, which is shifted outward compared to A_2A_AR, due to the shorter ECL3, andTM6 slightly shifted inward in A_1_AR. These rearrangements, in turn, affect the orthosteric site of A_1_AR, which is wider than A_2A_AR. Interestingly, the key residues in the orthosteric site of the two receptors are conserved and drive the same binding mode of the antagonist PSB36 (Figure 1). More precisely, the xanthine scaffold forms two hydrogen bonds with Asn254 (A_1_AR, Asn253 in A_2A_ AR) and a π- π stacking with Phe171 (A_1_AR, Phe168 in A_2A_AR). Nevertheless, Asn254 (A_1_AR) is located in the binding site deeper than Asn253 in A_2A_AR, and the xanthine ligand is consequently positioned deeply in the orthosteric site (Figure 1).

Despite the huge help provided from high-resolution structural biology techniques, certain selectivity profiles cannot be only rationalized by the mere coordinates of bound state or “final” state. Ligand recognition is an articulated mechanism in which many variables may play a relevant role and over the last few years, there has been rising attention in the understanding of binding kinetics at GPCRs and its determinant role to successfully target this class of proteins [19].

**Figure 2:**
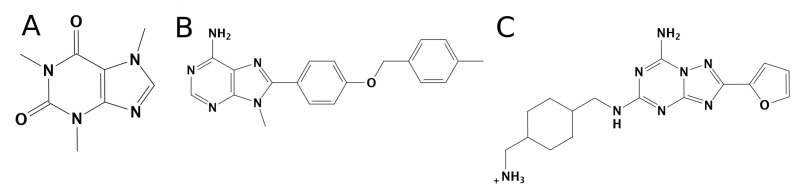
The three ligands considered in the present study. (**A**) Caffeine, a non-selective ARs antagonist. (**B**) LC4, an A_1_-AR selective antagonist. (**C**) Z48, an A_2A_-AR selective antagonist.

In the present study, we used supervised molecular dynamics (SuMD) simulations toshed light on the molecular basis of the selectivity of three different ligands to A_1_AR and A_2A_AR, not only considering the bound states, but also the possible different recognition mechanism preceding the final orthosteric site and the role of the solvent. We focus our attention on three antagonists: the A_2A_AR selective antagonists Z48 (Ki 16.9 nM in A_2A_AR and 1345.7 nM in A_1_AR) [20]; the A_1_AR selective antagonist LC4 (Ki 16,800 nM in A_2A_AR and 89 nM in A_1_AR) [21]; and the nonselective antagonist caffeine (Figure 2). SuMD [22,23] is a molecular dynamics (MD) approach that allows for the study of molecular recognition processes in a fully atomistic way, in the nanosecond timescale, without introducing any energetic biases.

## 2. Materials and Methods

### 2.1. System Setup

The crystal structures of the two receptors were retrieved from PDB (the PDB code is 5N2S for A_1_-AR and 5NM4 for A_2A_-AR). Systems preparation was performed using a Molecular Operating Environment (MOE)) suite (Chemical Computing Group ULC, Molecular Operating Environment (MOE), 2019.01. 1010 Sherbrooke St. West, Suite #910, Montreal, QC, Canada, H3A 2R7, 2019) [24] for protein preparation (removal of crystallographic water molecules, ions, and other solvent molecules, selection of the highest occupancy for each residue, assignment of the correct protonation state at pH 7.4). Systems preparation for the molecular dynamics simulations was carried out using VMD [25]. The protein was explicitly solvated in a water box with the borders placed at a distance of 15 Å from any protein atom, the water model used was TIP3P [26]. The system charge was neutralized to a concentration of 0.154 M using Na^+^/Cl^−^. The lipid bilayer consisted of phosphatidylcholine (POPC) units.

The sodium ionwithin the TMD allosteric site of A_2A_-AR was retained, andit was also placed by superposition in A_1_-AR.

### 2.2. Equilibration of the System

All the simulations were performed with a CHARMM36 force field [27] and using ACEMD2 [28]. Ligands parameters were retrieved from Paramchem [29], a web interface for the assignment of parameters based on the CGenFF [30] force field.

The system energy was minimized in 1500 steps using the conjugate-gradient method, then the equilibrationof the system was done in four steps. The first one consistedof 5 ns of NPT simulation with harmonic positional constraints of 1 kcal mol^−1^Å^−2^ on each atom of the protein and the lipid bilayer. The second step consisted in 10 ns of NPT simulation with harmonic positional constraints of 1 kcal mol^−1^Å^−2^ only on eachprotein atom and on the phosphorus atom of the POPC units, the third step consisted in 5 ns of NPT simulation with harmonic positional constraints of 1 kcal mol^−1^Å^−2^ only on the alpha carbons of the protein, and the last step consisted in 50ns of NVT simulation without any constraints.

For the productive simulations, the temperature wasmaintained at 310 K using the Langevin thermostat, with a low dumping of 1 ps^−1^. The pressure was set at 1 atm using the Berendsen barostat [31]. The particle-mesh Ewald (PME) method was used to calculate the electrostatic interactions with a 1 Å grid [32]. A 9.0 Å cutoff was applied for long-term interactions.The M-SHAKE algorithm was applied to constrain the bond lengths involving hydrogen atoms.

At the end of the equilibration, several parameterswere calculated to assess the stability of the system:theroot mean square deviation (RMSD) of the alpha carbons of the protein, the root mean square fluctuation (RMSF) of each protein residue, the volume of the cell (which should tend to a plateau in the NPT ensemble), and the area per lipid (APL) for each membrane layer (calculated using GridMAT-MD [33]). We also computed the volume of the orthostericsiteduring the equilibration using POVME [34]. Figure S1 (A_2A_-AR) and Figure S2 (A_1_-AR) report the analysis performed during the equilibration of the system. In both the two systems, the protein reached a stable conformation (RMSD of the protein Cα (panel A, Figures S1 and S2) stably below 2 Å for A_2A_-AR and below 3 Å for A_1_-AR). The volume of the orthosteric site reached a plateau in both cases (Panel B, Figures S1 and S2). The most flexible parts of the protein, as expected, are the loops. Indeed, the RMSF of these regions is higher than the TMs (panels C and D, Figures S1 and S2).

As shown in Figure S5, the orthosteric site of A_1_-AR appears to be deeper than A_2A_-AR, due to a cleft between TM5 and TM6. The APL and the volumetric analysis are reported in Figure S3 (A_1_-AR) and Figure S4 (A_2A_-AR). For both systems, the cell volume reached stable values.

### 2.3. Supervised Molecular Dynamics Simulations

**Figure 3:**
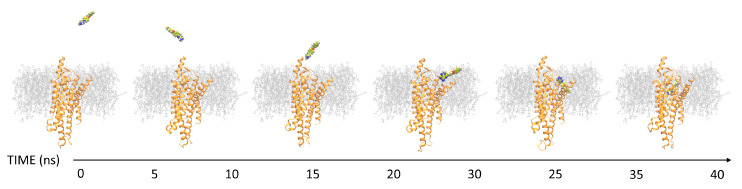
Representation of a binding event sampled by a supervised molecular dynamics (SuMD) simulation. After each reported step, the distance between the ligand and the binding site decreases. In less than (merged) 50 ns, a binding event was sampled.

SuMD [22,23] is a molecular dynamics (MD) approach that allows for the investigation of molecular recognition processes in a fully atomistic way, in the nanosecond timescale, without introducing any energetic bias (Figure 3). Ligands were placed 35 Åaway from the protein. EachSuMD step was set to 600 ps. During each SuMD step, the distance between the center of mass of the binding site (defined by a series of residues) and the center of mass of the ligand is monitored. These dataare thenfitted, and if the slope of the interpolating linear function is negative, then the coordinates and the velocities are used for the successive time window, otherwise, the last time window is simulated again reassigning the velocities (this reassignment of the velocities is intrinsic in the use of Langevin thermostat). If the condition fails 30 consecutivetimes, then the simulation is stopped. Otherwise, the algorithm continues until the distance between the two centers of mass is below the threshold of 5 Å; at this point, the supervision is turned off and 30 short classical MD simulations are performed, switching on the supervision if the distance between the centroids becomesgreater than 5 Å. At the end of the SuMD process, the trajectoryis prolonged for 25 ns of classical MD simulation.

Twenty simulations were performed for each of the six systems (Z48/A_1_AR, Z48/A_2A_AR, LC4/A_1_AR, LC4/A_2A_AR, Caffeine/A_1_AR, Caffeine/A_2A_AR). Only the simulationsthat sampled the ligand reaching the orthosteric site and interacting with the classic fingerprint of these class of ligands (Figure S6) were here reported (e.g., one replica for each system, excepted LC4/A_1_AR for which two replicas were analyzed). For each system, the reasonsfor failure are similar. In most cases, the ligands interact strongly with the residues of the ECLs, and do not reach the binding site. In a few cases, the ligands only partially reach the orthosteric site. Finally, in other rare cases, the ligands get stuck between the protein and the membrane.

### 2.4. Trajectories Analysis

The SuMD trajectories were analyzed using anin-house python tool (described in [35]) that provides information on the geometry and the energetic of the system. The output consists of a per-residue analysis of the electrostatic and van Der Waals contributions to the protein-ligand interactions; a representation of the distance between the center of mass of the ligand and the center of mass of the binding site as a function of time; a global energetic evaluation of the system as a function of the aforementioned distance. This analysis allowscomparing the energetic profile of two systemsboth in a general way and, through the per-residue analysis (i.e., it is possible to evaluate which ligand has better interaction with some specific amino acids of interestover the time).

Three replicas of 50 ns were performed on the apo form of A_1_-AR and A_2A_-AR, after the equilibration stage described before. These three replicas were merged and analyzed by AquaMMapS [36].

## 3. Results

### 3.1. SuMD Binding of the A_1_AR Nonselective Antagonist Caffeine

According to the SuMD simulations (Figures S7 and S8; Video S1 for A_1_AR and Video S2 for A_A2A_AR in the Supplementary Information), the nonselective antagonist caffeineestablishes intermediate interactions with the extracellular vestibule of A_1_AR and A_2A_AR, before reaching the orthosteric site. The most stable bound configurations sampled on A_1_AR and A_2A_AR differed for theorientation of the xanthine ring (Figure S9). On A_2A_AR, caffeine pointed the N7-methyl toward Asn253, while in A_1_AR it was rotated by 180°, with the N3 methylinthe proximity of Asn254. Notably, both these two conformations have been experimentally observed in X-ray crystal structuresofA_2A_AR [17]. Fluctuations of the ligand in both orthosteric sites and the transient nature of the interactions are easily depicted in the energy interaction landscapes (Panels A,S7,S8) in which the points are particularly scattered over the distance between the centers of mass of the ligand and the orthosteric site.

### 3.2. SuMD Binding of the A_2A_AR Selective Antagonist Z48

Figures S10 and S11 reportthe analysis of Z48/A_1_ARand Z48/A_2A_AR, including 25 ns of classic MD simulations performed at the end of each SuMD simulation (Videos S3 and S4 in the Supplementary Information).

**Figure 4:**
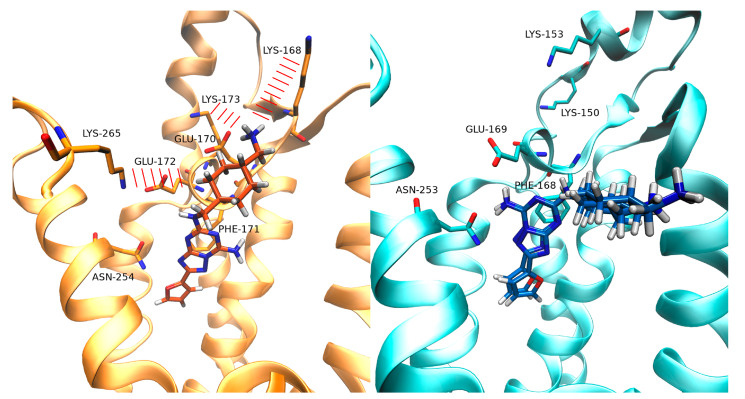
Binding mode of Z48 within the binding site of A_1_-AR on the left-hand side, and within the binding site of A_2A_-AR on the right-hand side (superposition of the two simulations analyzed). For A_1_AR, the binding mode is reported at the end of the SuMD simulation. For A_2A_AR, the two poses at the end of the classic MD simulation are reported.

For what concerns the SuMD trajectory of Z48 on A_1_AR, notable electrostatic repulsion took place between the ligand and Lys168, Lys173, and Lys265, before the ligand reached the orthosteric site, as clearly observable in the per-residue electrostatic interaction energy plot (Panel C,S10). These residues are positioned on the ECLs (Lys168 and Lys173 on ECL2, andLys265 on ECL3). As a result, in A_1_AR, Z48 did not adopt the binding fingerprint of the ARs antagonists (e.g., only one hydrogen bond with Asn254 out of two was formed). Moreover, this binding mode wasunstable over the 25 ns of classic MD simulations.Interestingly, the terminal amine group of the ligand strongly interacts with Glu170 (Leu167 in A_2A_AR). Figure 4 reports the binding modes of Z48 at the end of the SuMD simulation on A_1_AR (the binding mode at the end of the 25 ns of classic MD simulation is shown in Figure S12) and A_2A_-AR (at the end of the 25 ns of classic MD simulation), respectively.

Moving to the binding simulations of Z48 toA_2A_AR, two SuMDreplicas led to the classic binding mode of the ARs antagonists. The π-π stacking with Phe168 was present, along with the hydrogen bonds with Asn253 and with Glu169. Both binding modeswere stable during 25 ns of classic MD simulations (Figure S11, Panel C,D, Figure 4). No significantprotein-ligand electrostatic repulsionswereobserved. The narrow funnel-like interaction energy profile of the ligand in the orthostatic also suggested a good complementarity of the ligand in the pocket, and a rapid reaching of a stable bound state.

### 3.3. SuMD Binding of the A_1_AR Selective Antagonist LC4

**Figure 5:**
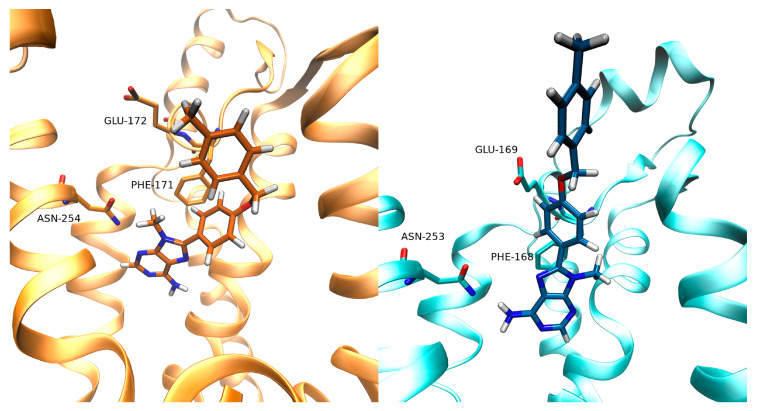
Binding mode of ligand LC4. A_1_AR on the left and A_2A_AR on the right.

**Figure 6:**
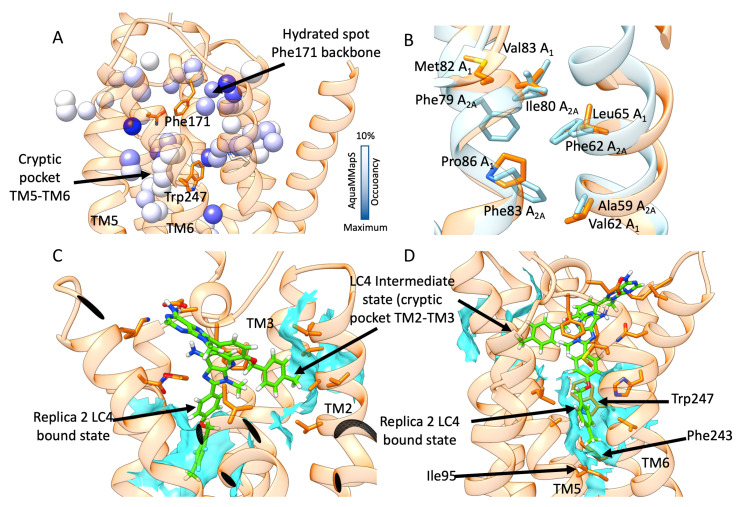
(**A**) Hydrated spots within A_1_AR with AquaMMapSoccupancy > 10%. The cryptic pocket between TM5 and TM6, as well as the spot nearby the Phe171 backbone, are indicated; (**B**) comparison between A_1_AR (orange) and A_2A_AR(cyan) at the level of TM2 and TM3—a sub-pocket formed only in A_1_AR during MD simulations, due to different residues and interhelical packing; (**C**) and (**D**) two side views of the two superimposed binding steps of LC4 (green stick) to A_1_AR. Hydrophobic contacts are shown as cyan transparent surfaces.

With regards to LC4, two SuMDreplicas led to different binding modes of LC4 into the A_1_AR orthosteric site. During Replica 1, the ligand formed a complex characterized by the xanthine scaffold positioned in the orthosteric binding site, and the N8-substituent pointed outward the receptor (Figure 5). A hydrogen bond with Asn254 and hydrophobic contacts with Phe171 occurred (Figure 6A). Interestingly, the LC4 oxygen atom in the N8 substituent interacted with and stabilized water molecules in the proximity of the Phe171 backbone, a hydrated spot on ECL3, according to the AquaMMapS analysis (Figure 6A).

In the case of A_2_AR, the xanthine scaffold reached the orthostericsite rotated by 180° compared to the binding mode adopted in A_1_AR (Figure 6B) and with an unfavorable geometry for hydrogen bonding with Asn253. In both of these two binding modes (Figures S13 and S14), LC4 did not interactwithconserved glutamate Glu172 (A_1_AR) or Glu169 (A_2A_AR).

During SuMDReplica 2 on A_1_A, LC4 experienced a two-step binding (Figure 6). First, the antagonist entered the orthosteric site pointing the methylphenyl group into a hydrophobic pocket located between TM2 and TM3. This cryptic pocket is not present in A_2_AR in light of bulkier residues and a higher degree of packing between the helixes (Figure 6B). From this metastable configuration, LC4 moved deeper into the orthosteric site and engagedAsn254 and Glu172 in hydrogen bonds (Figure 6). In this binding mode, the ligand inserted the N8-substituent inside a further cryptic pocket between TM5 and TM6 (Figure 6C,D), which is delimited by the “toggle switch” residue W247 [15], and the residues Ile95 and Phe253, being part of the conserved class A structural motif PIF [37]. Notably, this hydrophobic sub-pocket was occupied by likely “unhappy” water molecules during simulations of the apo-A_1_R (Figure 6A).

The video of these three simulations can be found in Supplementary Materials Video S5 and Video S6 for the two simulations of LC4/A_1_AR, and Video S7 for the simulation of LC4/A_A2A_AR.

## 4. Discussion

Here we present results from SuMD simulations performed to shed light on the selectivity displayed by LC4 and Z48, two antagonists of A_1_AR and A_2A_AR, respectively. The nonselective antagonist caffeine was also dynamically docked to the two ARs subtype.Caffeine, which is in a weak binder(micromolar range [38]) of all the ARs, experienced more than one binding mode, in line with our previous simulations [22] and experimental observation [17].

SuMD binding of the selective A_2A_AR antagonistZ48 on A_1_-AR and A_2A_AR suggested different interaction patterns along the pathways. Z48 experienced unfavorable electrostatic interactions between positively charged A_1_AR residues Lys168, Lys173 (ECL2), and Lys265 (ECL3), and the ligand charged amine on the N8-substituent. Such transitory states did not take place during binding to A_2A_AR, as no significant electrostatic repulsions were computed at the extracellular vestibule. Interestingly, A_2A_AR bears Ala265 instead of Lys265 on the ECL3, while Lys168 and Lys173 (ECL2) are farther from the binding site, compared to A_1_AR. This is consistent with mutagenesis studies that demonstrated the importance of these lysine residues forthe binding of several A_1_AR ligands [39,40,41]. On A_2A_AR, Z48 reachedthe orthosteric site producing the classic interactions fingerprint of the ARs antagonists. On A_1_AR, on the other hand, the ligand sampled a different binding mode. Moreover, the A_1_AR residue Glu170 (Val167 in A_2A_AR) strongly interacted with the charged terminal amine of the ligand, stabilizing this alternative binding mode. Z48 was proposed to bind to A_2_AR overcoming low enthalpy transition state(s) [40]. From this standpoint, the unfavorable electrostatic interactions with ECL2 could implicate a slower binding toA_1_AR, and therefore a kinetic selectivity for A_2_AR.

SuMD simulations of the antagonist LC4 proposed two possible binding modes that could drive selectivity. It is possible that the ligand binds A_1_AR and A_2A_AR with the same conformation, but differently interacting with water molecules nearby ECL3. From this standpoint, and in analogy with [41], we propose these “happy” water molecules contribute to the ligand stabilization in A_1_AR (Figure S16), but not in A_2_AR (Figure S17). An alternative and unique LC4 binding mechanism was sampled only on A_1_AR, with a metastable state before the final complex formation. Along this pathway, two cryptic hydrophobic pockets (between TM2 and TM3 and between TM5 and TM6) allowed the N8-substituent of the ligand to correctly orient first, and then engage key residues for the receptor activation (the “toggle switch” Trp247, Ile95, and Phe253, which are part of the conserved class A motif PIF). Notably, the cryptic pocket between TM2 and TM3 has recently been proposed as a determinant for A_1_AR selectivity displayed by the triazolotriazine antagonist LUF5452 [42,43].

## 5. Conclusions

Understanding the selectivity of GPCRs ligands is an important task in drug design. This study supports the emerging idea that selectivity is driven by a plethora of phenomena, other than the protein-ligand interactions in the bound state. Receptor-ligands recognitions are multistep events modulated by intermediate interaction along withthe (un)binding paths. This picture may be further complicated by the presence of stable water molecules, whichcan have a tremendous impact on stabilizing or destabilizing an orthosteric complex. To consider different aspects that may affect the selectivity on A_1_AR and A_2A_AR, we used SuMD simulations to investigate the recognition of thee different antagonists. Overall, our results suggest that kinetic selectivity may favor the binding of Z48 to A_2A_AR over LC4. A different scenario was observed for A_1_AR, the recognition trajectories highlighted the key role of water molecules in the binding mode of LC4, which is favored by two hidden sub pockets within A_1_AR.

## Supplementary Materials

The following are available online at https://www.mdpi.com/2218-273X/10/5/732/s1, Figure S1: A_2A_AR, protein analysis during the equilibration stage, Figure S2: A_1_AR, protein analysis during the equilibration stage. Figure S3: A_1_AR system, cell volume analysis and membrane analysis. Figure S4: A_2A_AR system, cell volume analysis and membrane analysis. Figure S5: Volumetric analysis (using POVME) of the orthosteric site of A_1_AR and A_2A_AR. Figure S6: X-Ray structure of A_2A_AR in complex with caffeine and with ZMA. Figure S7: Analysis of the SuMD trajectory of binding for caffeine on A_1_AR. Figure S8: Analysis of the SuMD trajectory of binding for caffeine on A_2A_AR. Figure S9: Caffeine binding mode of caffeine within the binding site of A_1_AR and A_2A_AR. Figure S10: Analysis of the Z48 SuMD binding trajectoryto A_1_AR. Figure S11: Analysis of the SuMD trajectory of binding for Z48 on A_2A_AR. Figure S12: Binding mode of Z48 in A_1_AR at the end of the 25ns of classic MD. Figure S13 Analysis of the SuMD trajectory of binding for LC4 on A_1_AR. Figure S14 Analysis of the SuMD trajectory of binding for LC4 on A_2A_AR. Figure S15 Analysis of the SuMD trajectory of the alternative binding for LC4 on A_1_AR. Figure S16: Interaction between LC4 and water molecules inA_1_AR. Figure S17: Interaction between LC4 and water molecules in A_1_AR and A_2A_AR. Video S1: Caffeine binding pathway against A_1_-AR., Video S2: Caffeine binding pathway against A_2A_-AR, Video S3: Z48 binding pathway against A_1_-AR. Video S4: Z48 binding pathway against A_2A_-AR. Video S5: LC4 binding pathway against A_1_-AR, binding mode 1. Video S6: LC4 binding pathway against A_1_-AR, binding mode 2. Video S7: LC4 binding pathway against A_2A_-AR.
