# Supplementary material for: New Insights into Key Determinants for Adenosine 1 Receptor Antagonists Selectivity Using Supervised Molecular Dynamics Simulations
Source: Biomolecules. 2020 May 7;10(5):732. doi: 10.3390/biom10050732 (PMC7278174; doi:10.3390/biom10050732)
Supplement: Supplementary file 1 [file biomolecules-10-00732-s001.zip › biomolecules-778930-supplementary-final/biomolecules-778930-Figures.pdf]

New insights into Key Determinants for Adenosine 1 Receptor Antagonists Selectivity using Supervised Molecular Dynamics Simulations

Giovanni Bolcato [1], Maicol Bissaro [1], Giuseppe Deganutti [1], Mattia Sturlese [2] and Stefano Moro [2,*]

[1] Molecular Modeling Section (MMS), Department of Pharmaceutical and Pharmacological Sciences, University of Padova, via Marzolo 5, 35131 Padova, Italy
[2] School of Life Sciences, University of Essex, Wivenhoe Park, Colchester, CO4 3SQ, U.K
[*] Correspondence: stefano.moro@unipd.it

Supplementary Information

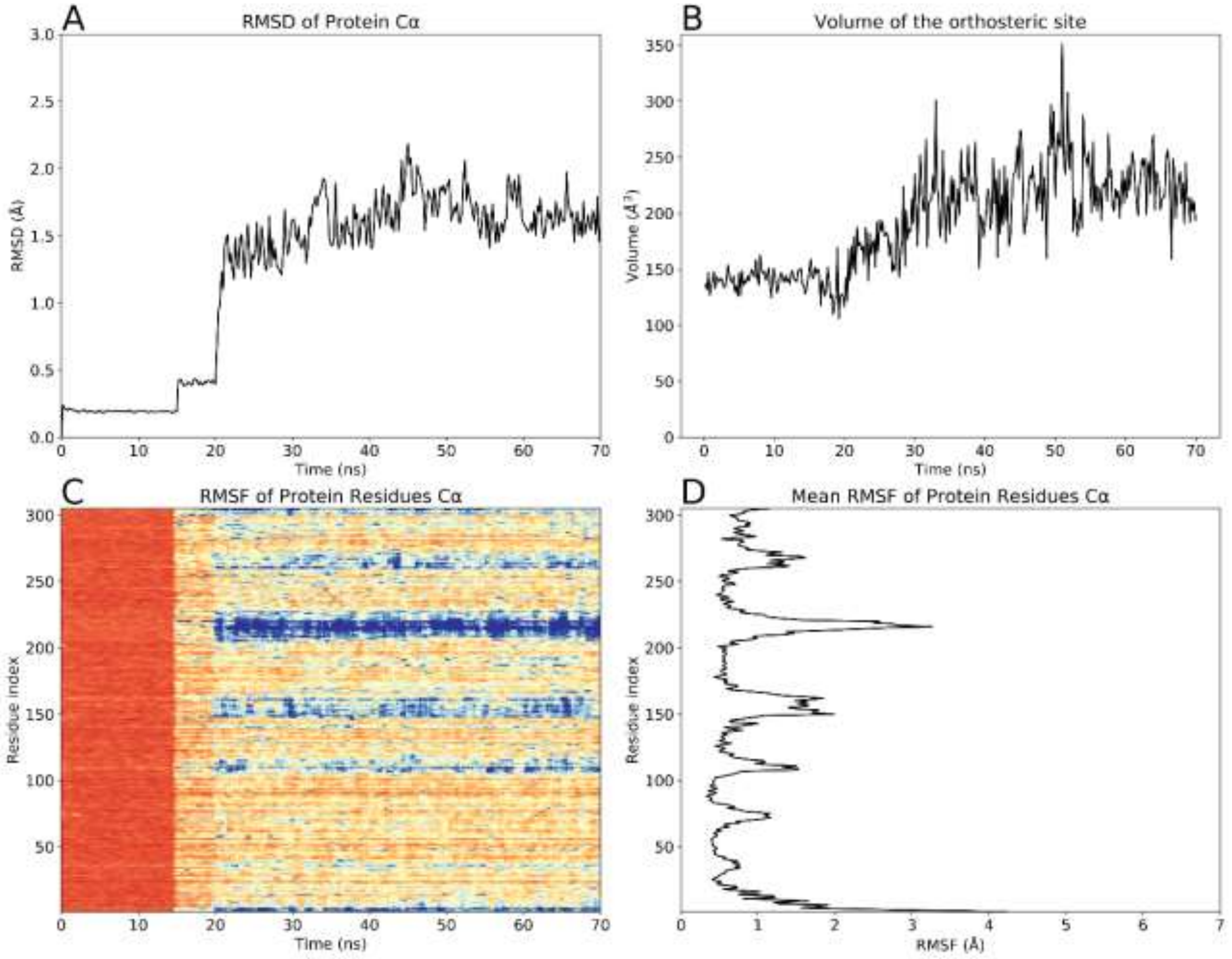

Figure S1 A_{2A}AR, protein analysis during the equilibration stage. Panel A: RMSD of the Protein Cα. Panel B: Volume of the orthosteric site. Panel C: RMSF of Protein residues Cα as a function of time, the RMSF value is plotted as a color, where blue indicates an higher value a red a lower value. Panel D: Mean RMSF of Protein residues Cα.

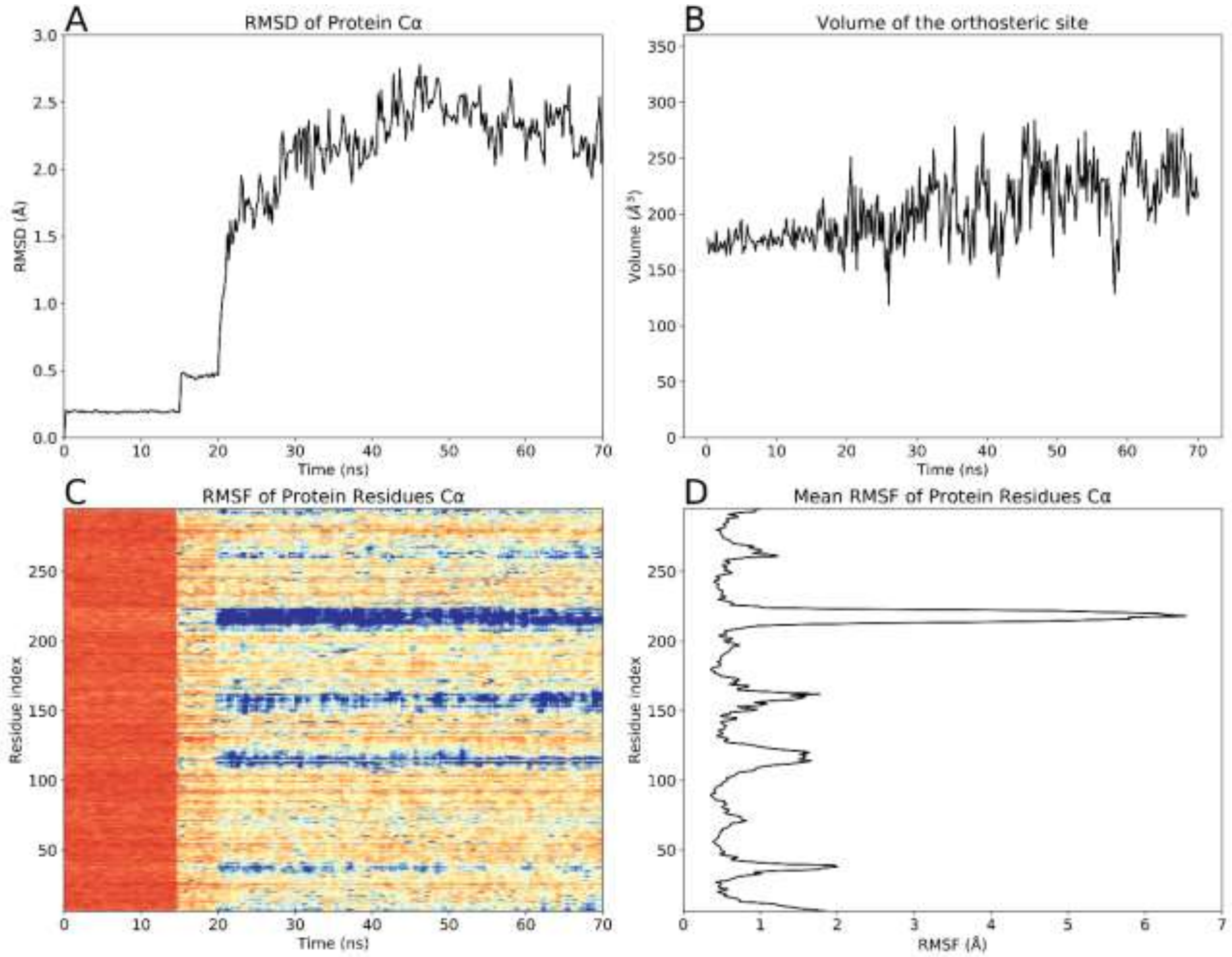

Figure S2 A_1AR, protein analysis during the equilibration stage. Panel A: RMSD of the Protein Cα. Panel B: Volume of the orthosteric site. Panel C: RMSF of Protein residues Cα as a function of time, the RMSF value is plotted as a color, where blue indicates an higher value a red a lower value. Panel D: Mean RMSF of Protein residues Cα.

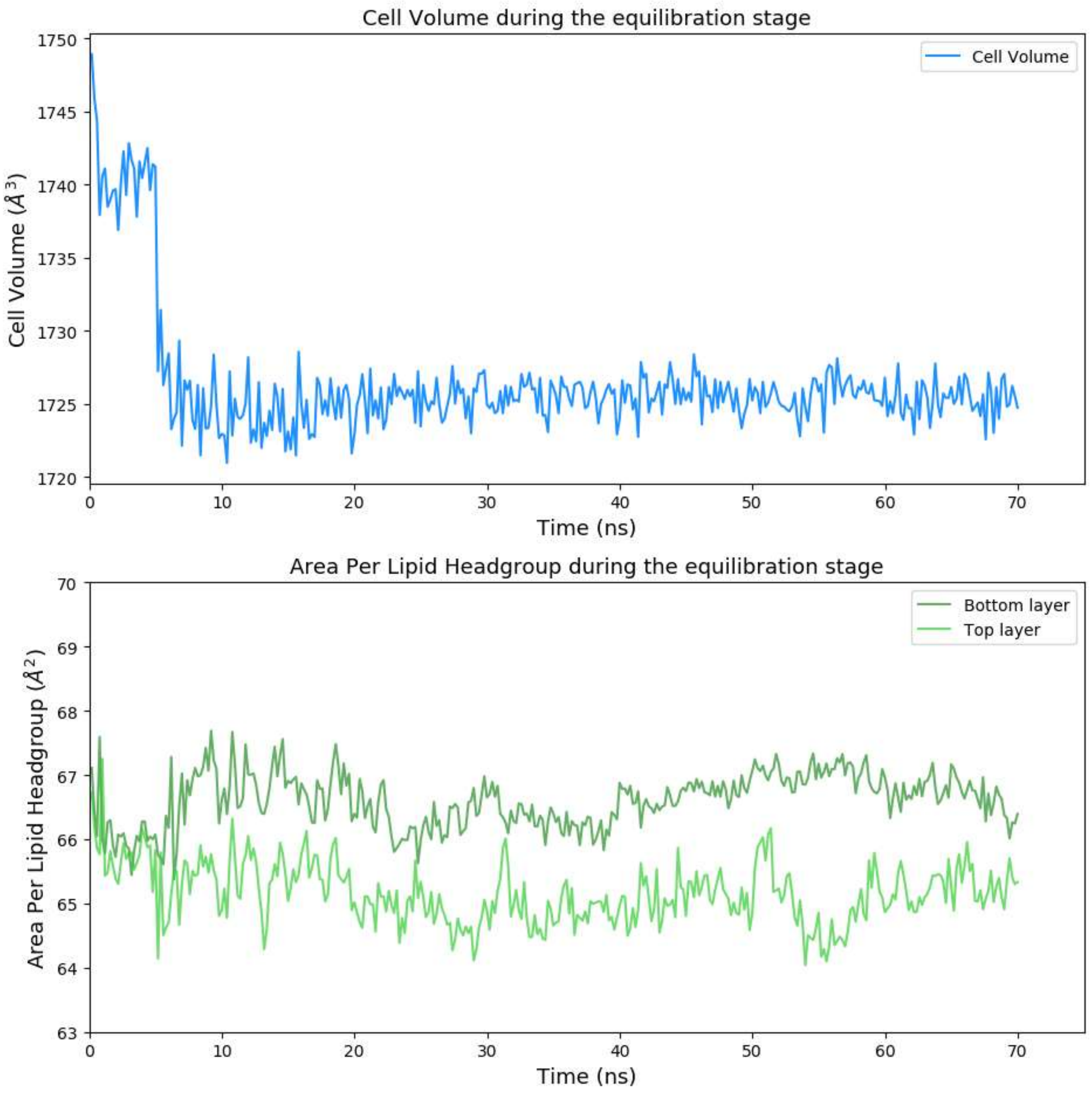

Figure S3 A_1AR system, cell volume analysis and membrane analysis. In the upper panel is reported the cell volume analysis. In the lower panel is reported the Area per Lipid Headgroup (analysis preformed with GRIDMAT).

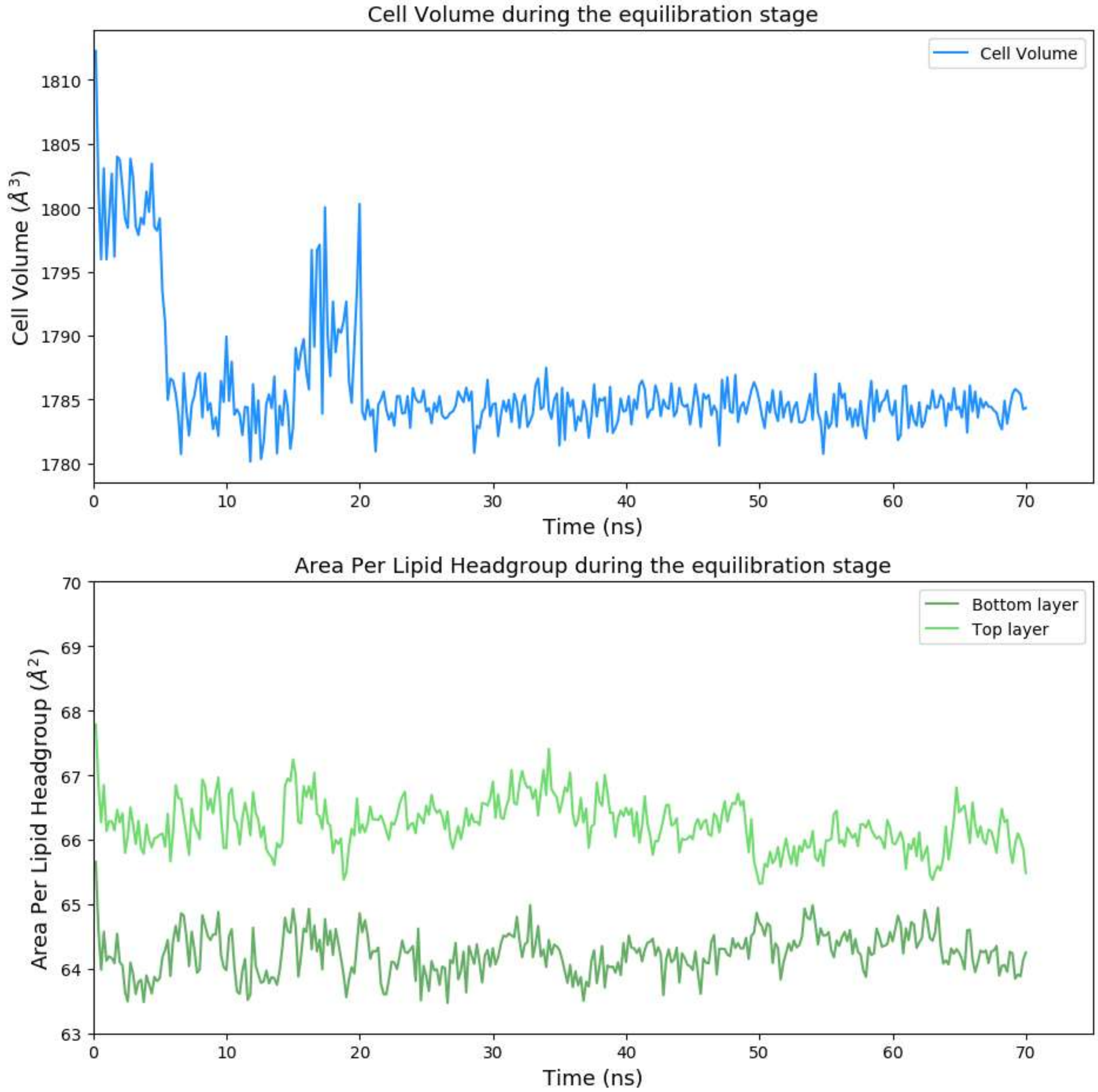

Figure S4 $A_{2A}AR$ system, cell volume analysis and membrane analysis. In the upper panel is reported the cell volume analysis. In the lower panel is reported the Area per Lipid Headgroup (analysis preformed with GRIDMAT).

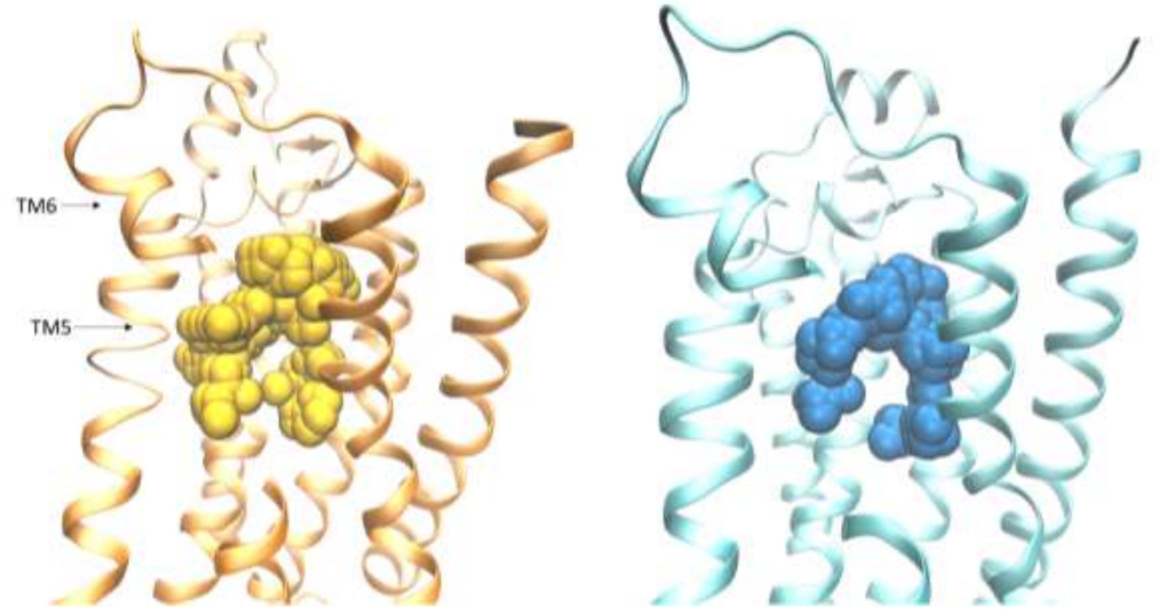

Figure S5. Volumetric analysis (using POVME) of the orthosteric site of A_1AR and $A_{2A}AR$. At the end of the equilibration stage, the orthosteric site of A_1AR is deeper than the one of A_{2A}-AR, especially between TM5 and TM6.

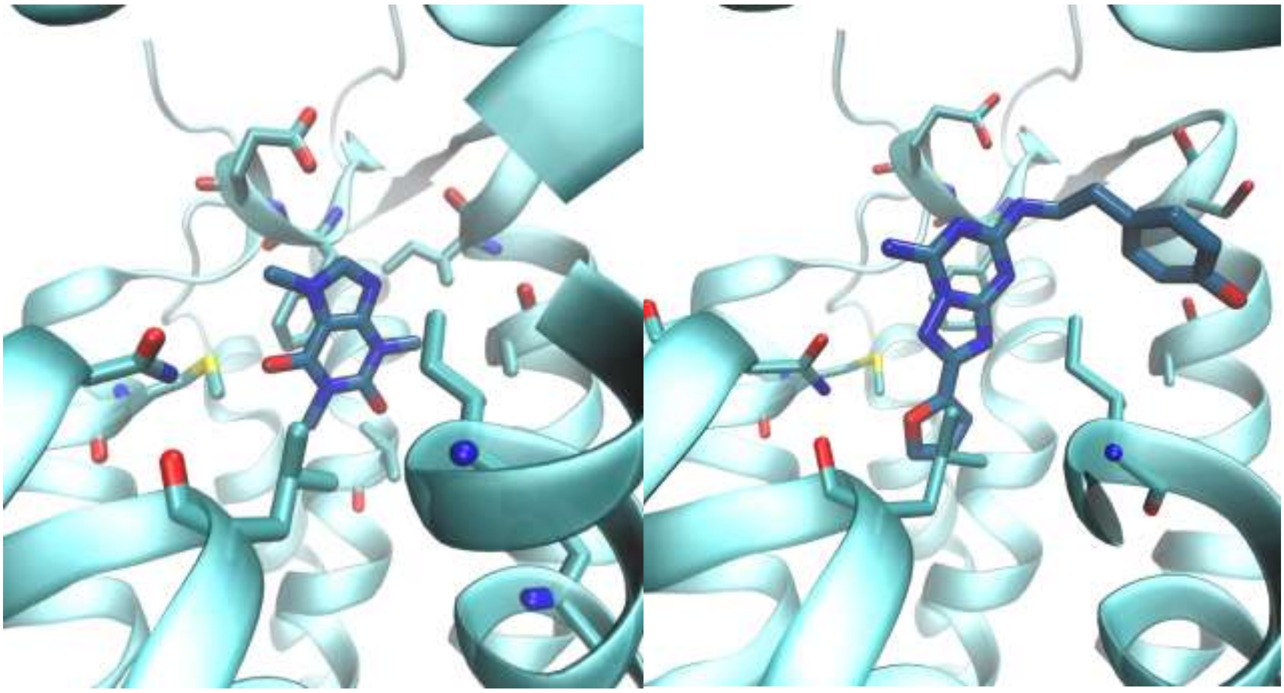

Figure S6. X-Ray structure of A_{2A}AR in complex with caffeine (left) and with ZMA (right)

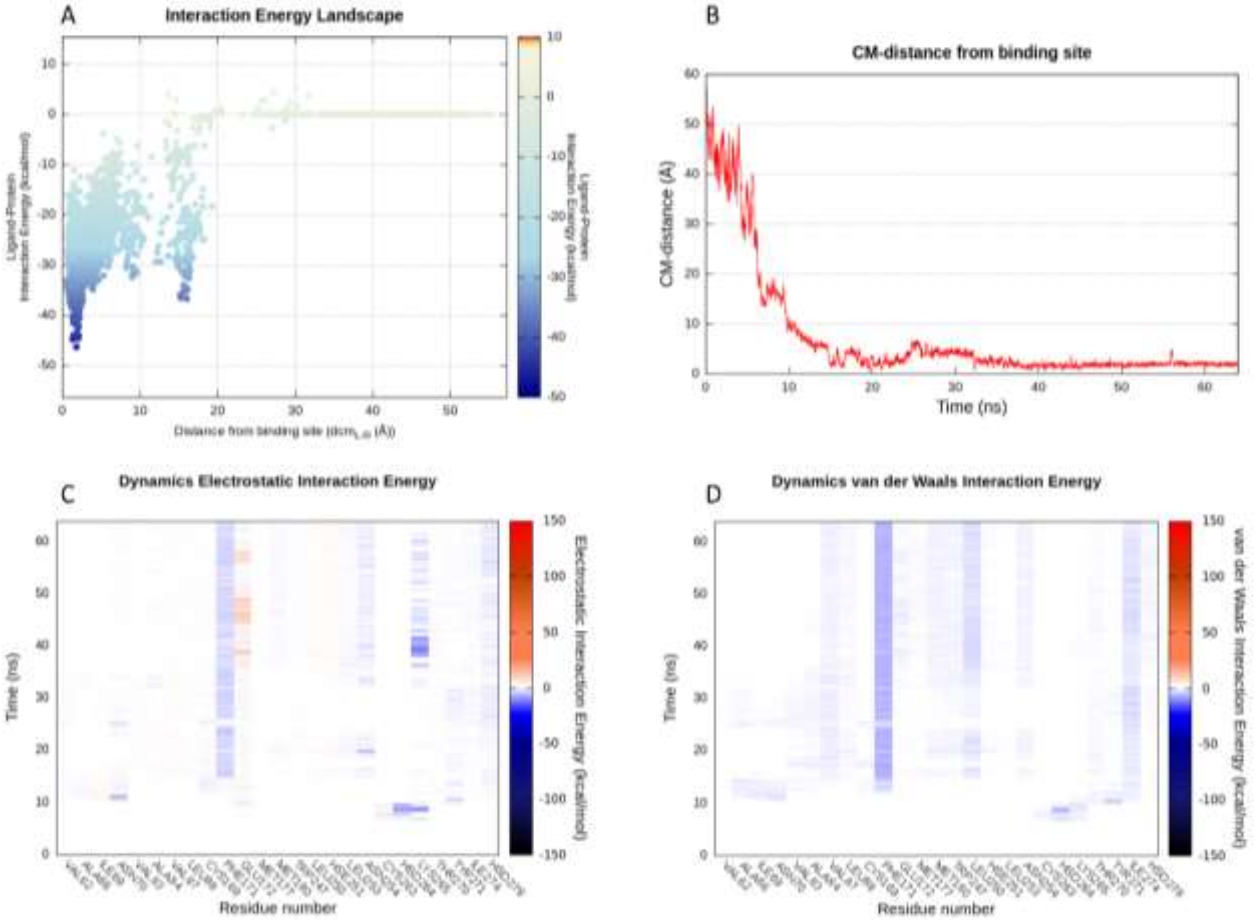

Figure S7 Analysis of the SuMD trajectory of binding for caffeine on A_1AR. Panel A: Ligand-protein interaction energy as a function of distance of the ligand from the binding site. Panel B: Distance between the center of mass of the ligand and the center of mass of the binding site over the time. Panel: Per-residue electrostatic interaction energy between ligand and protein. Panel D: Per residue van der Waals interaction energy between ligand and protein

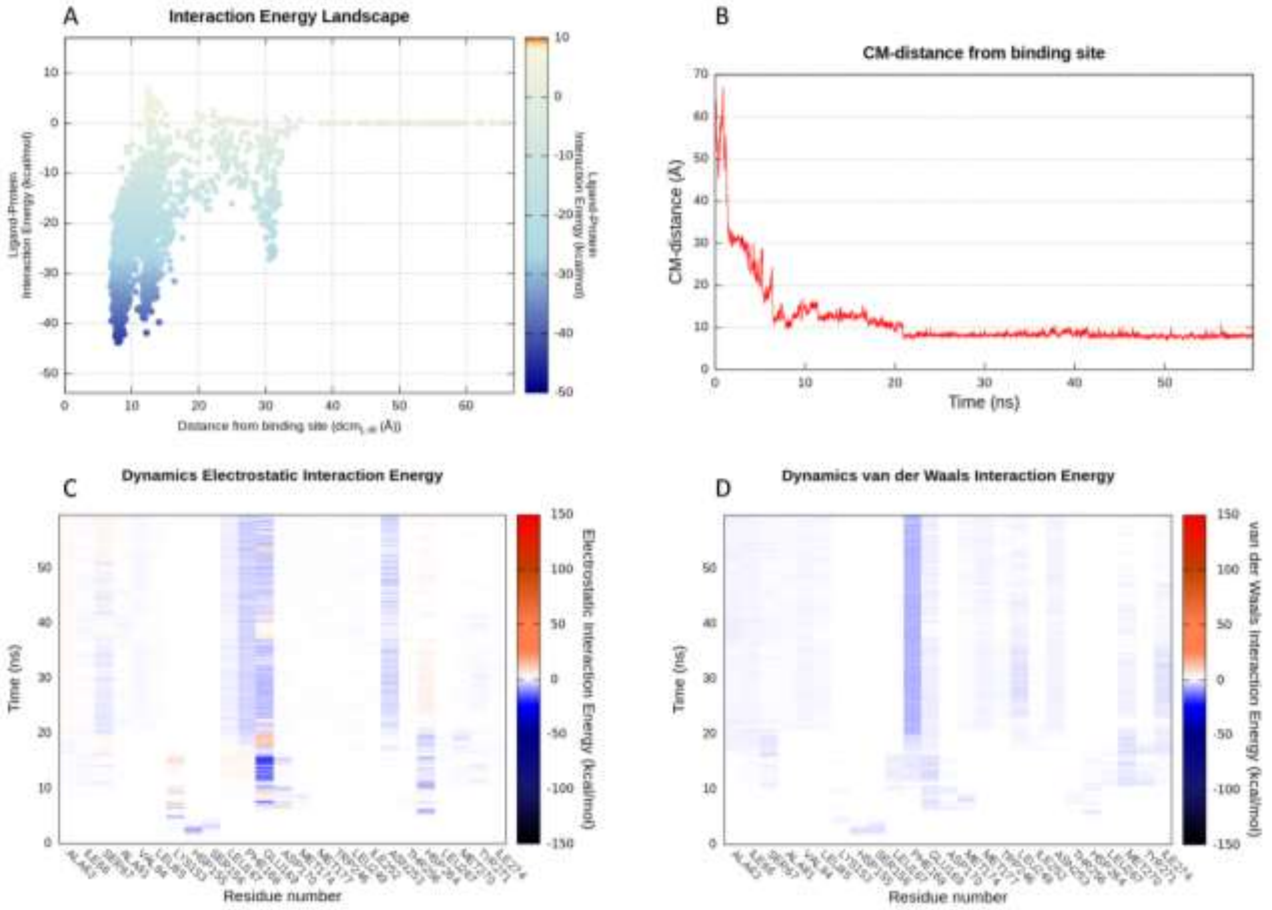

Figure S8 Analysis of the SuMD trajectory of binding for caffeine on $A_{2A}AR$. For a detailed explanation of each panel see Figure 5.

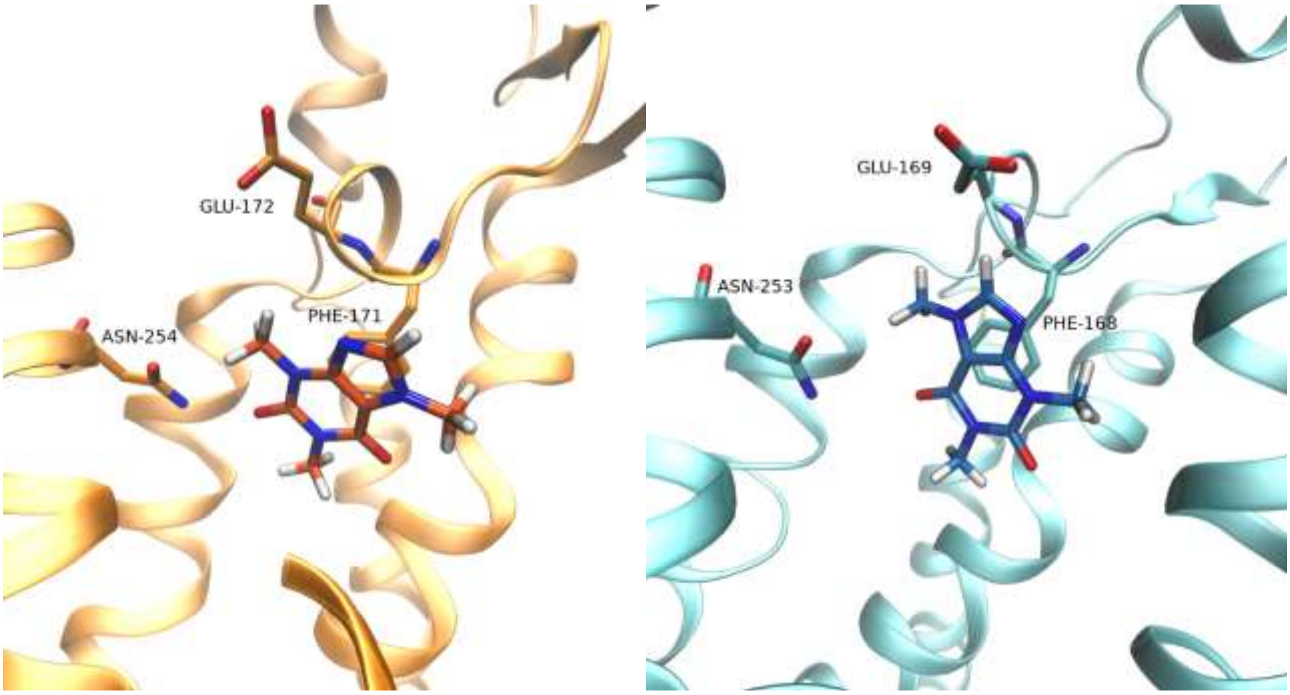

Figure S9 Caffeine binding mode of caffeine within the binding site of A_1AR, on the left-hand side, and within the $A_{2A}AR$ binding site, on the right-hand side.

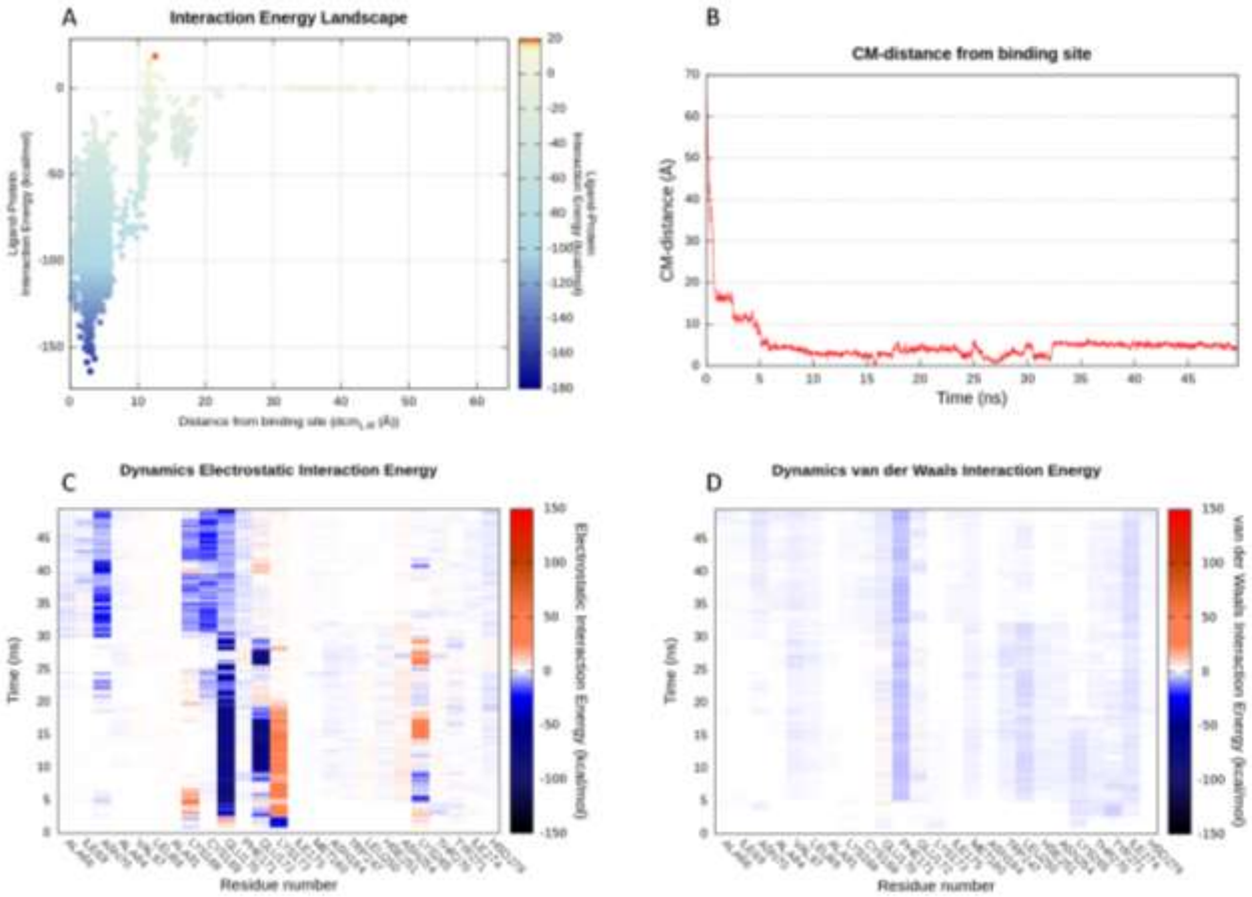

Figure S10 Analysis of the Z48 SuMD binding trajectory to A_1AR. For a detailed explanation of each panel see Figure S7

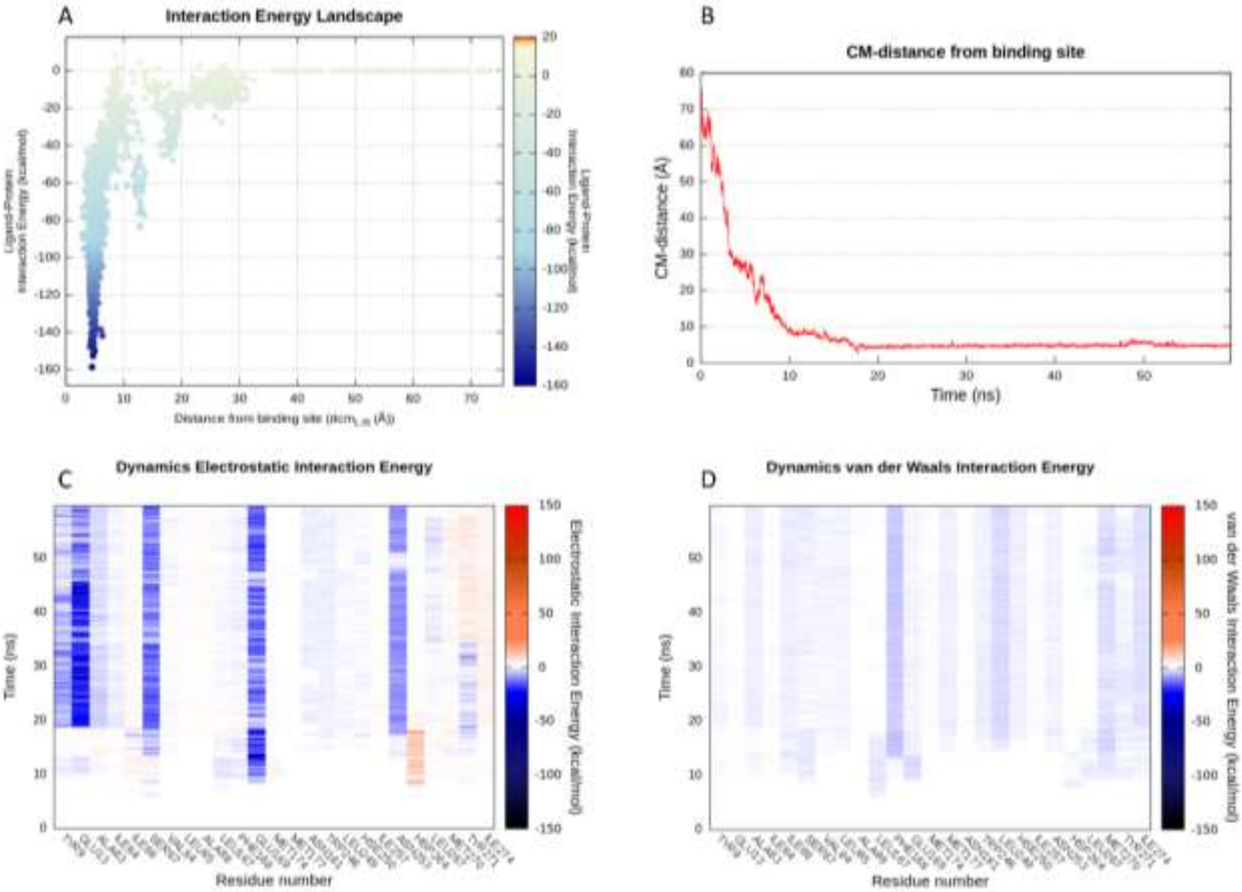

Figure S11 Analysis of the SuMD trajectory of binding for Z48 on A_{2A}AR. For a detailed explanation of each panel see Figure S7

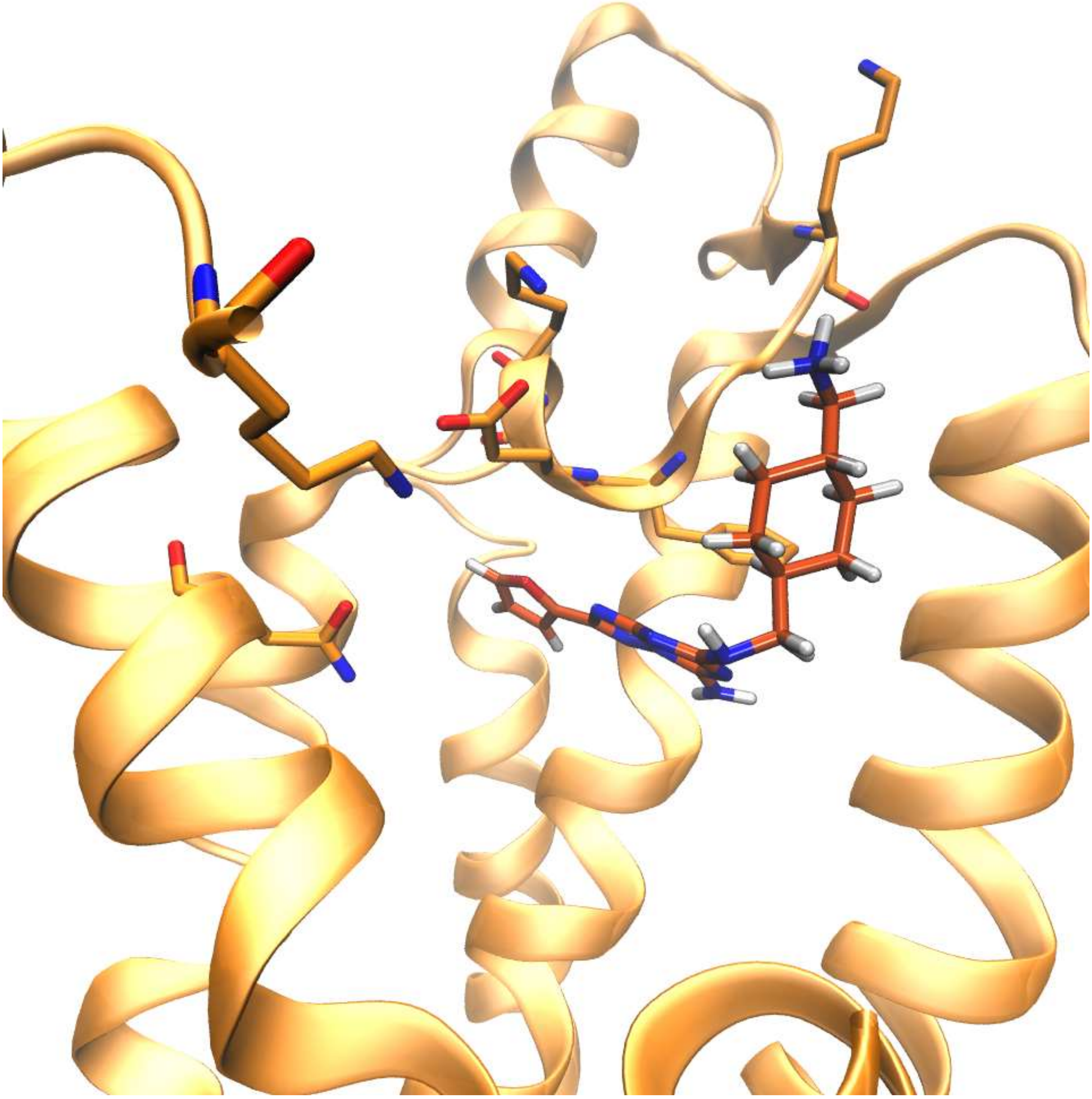

Figure S12 Z48 in A_1AR lose his binding mode in the 25 ns of classic MD performed at the end of SuMD.

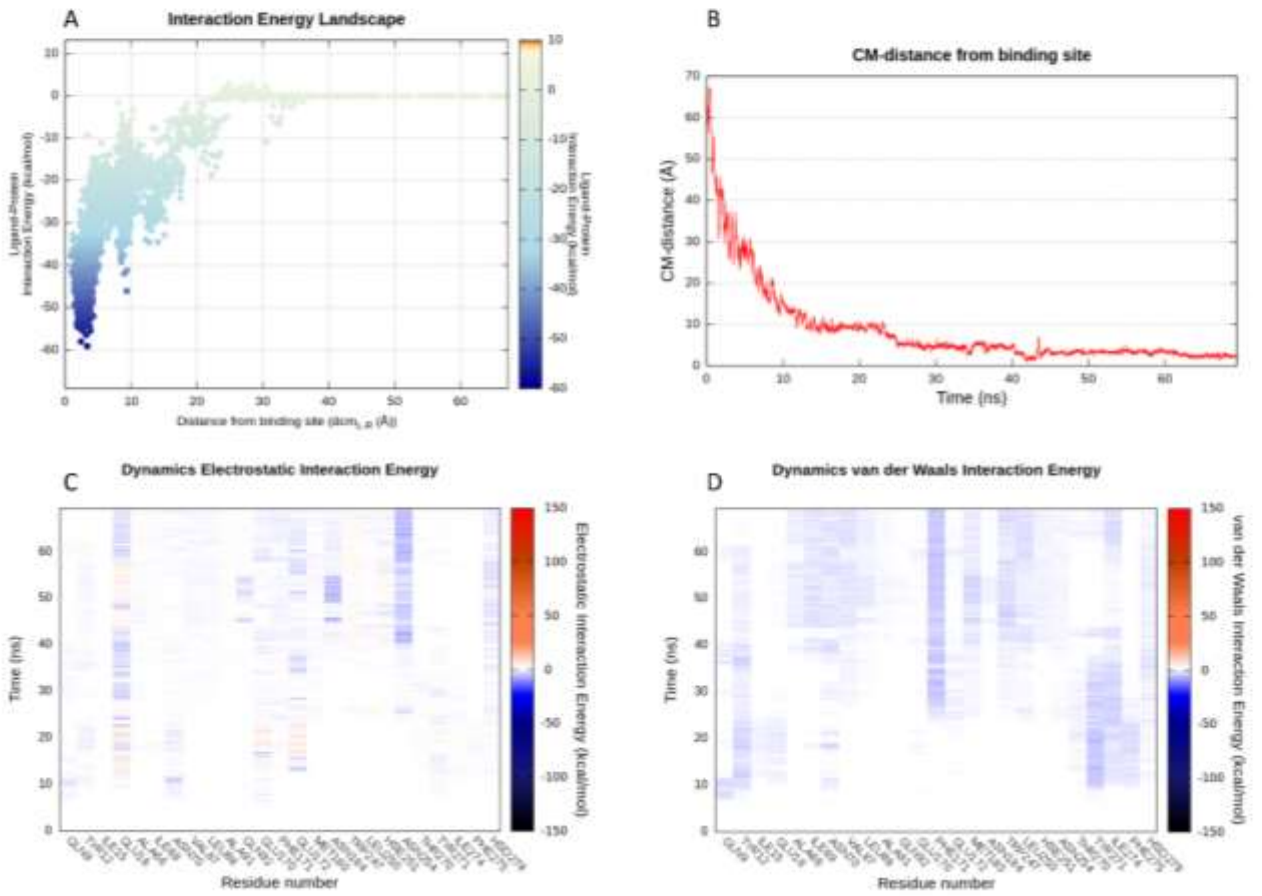

Figure S13 Analysis of the SuMD trajectory of binding for LC4 on A_1AR. For a detailed explanation of each panel see Figure S7.

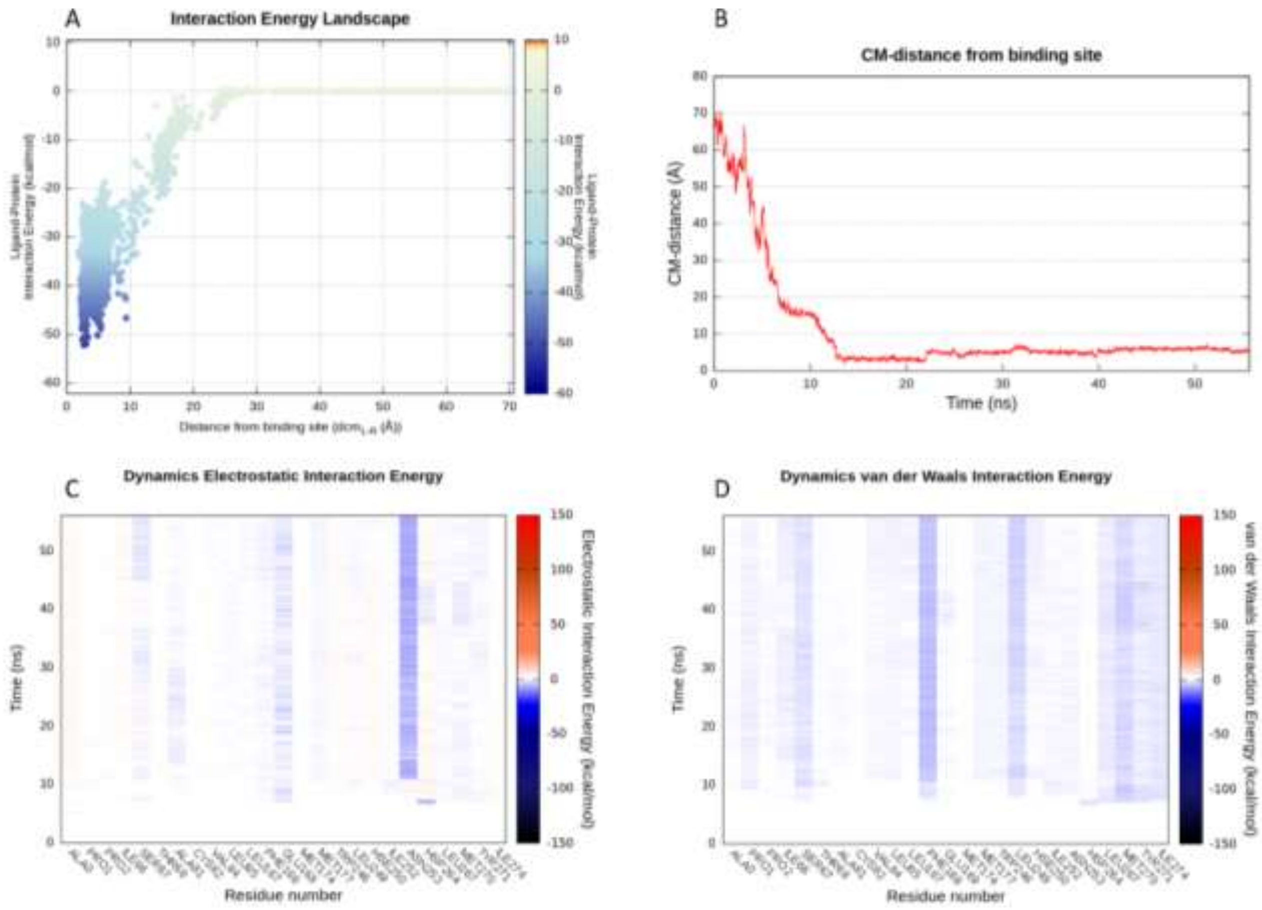

Figure S14 Analysis of the SuMD trajectory of binding for LC4 on A_{2A}AR. For a detailed explanation of each panel see Figure S7.

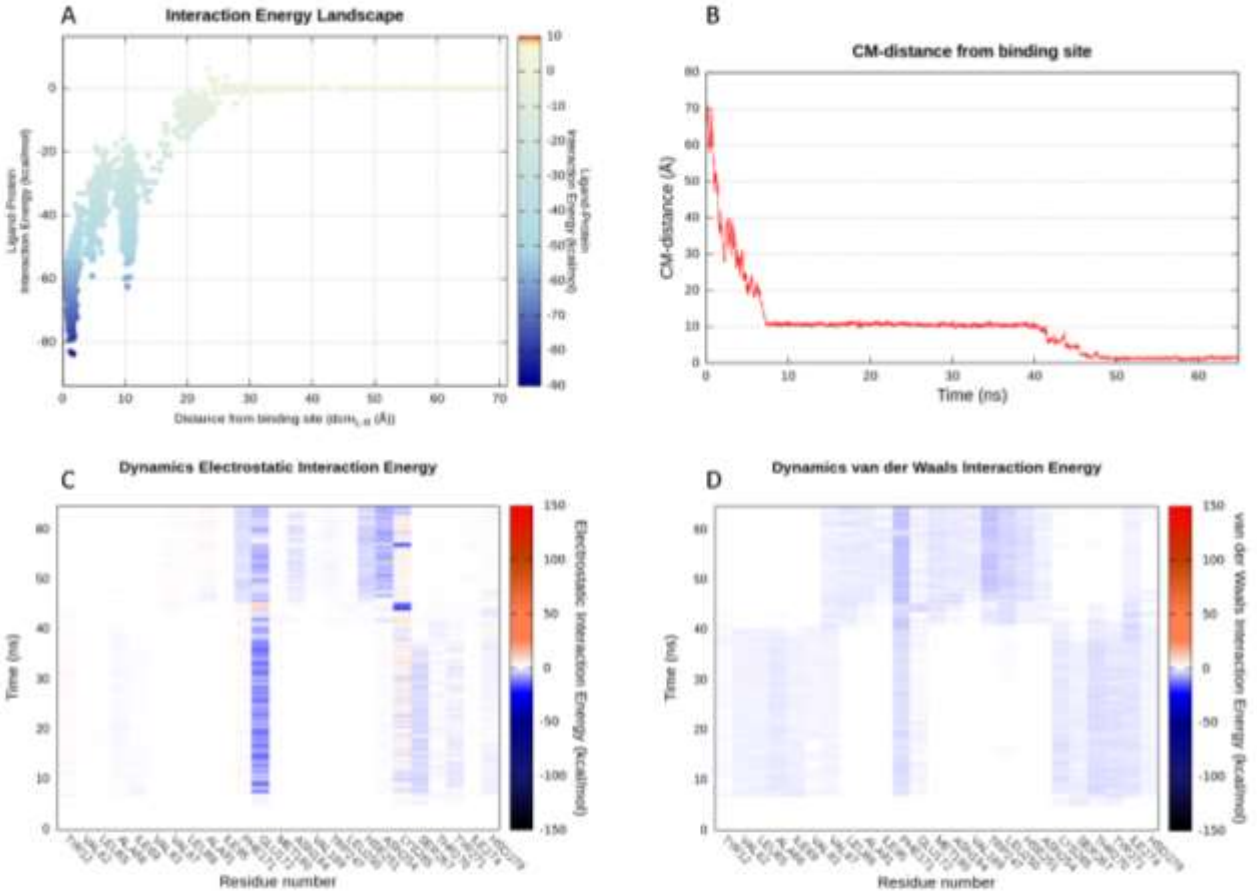

Figure S15 Analysis of the SuMD trajectory of the alternative binding for LC4 on A_1AR. As it can seen in panel C and D, at the end the ligand adopt the classical binding mode interacting with ASN-254, GLU-172 and PHE-171.

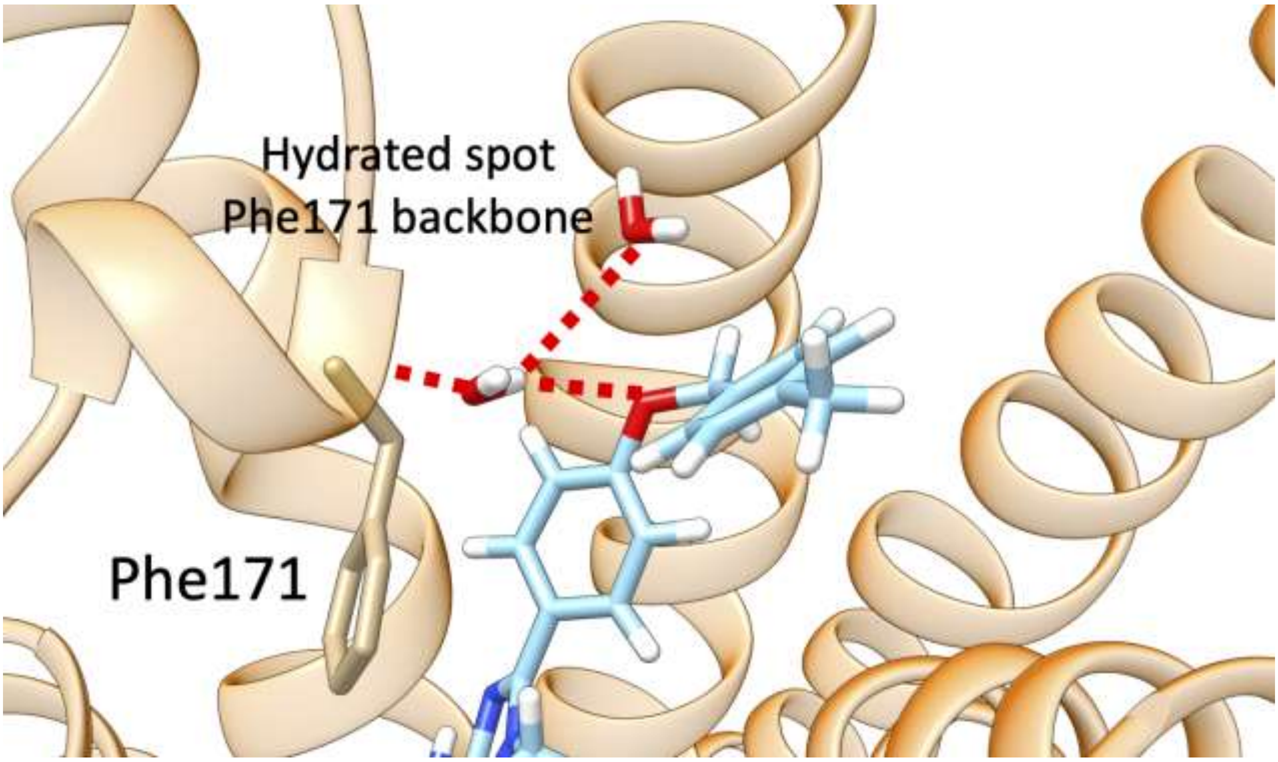

Figure S16 The favorable intraction between LC4 and the water spot near the backbone of Phe171 in A_1AR

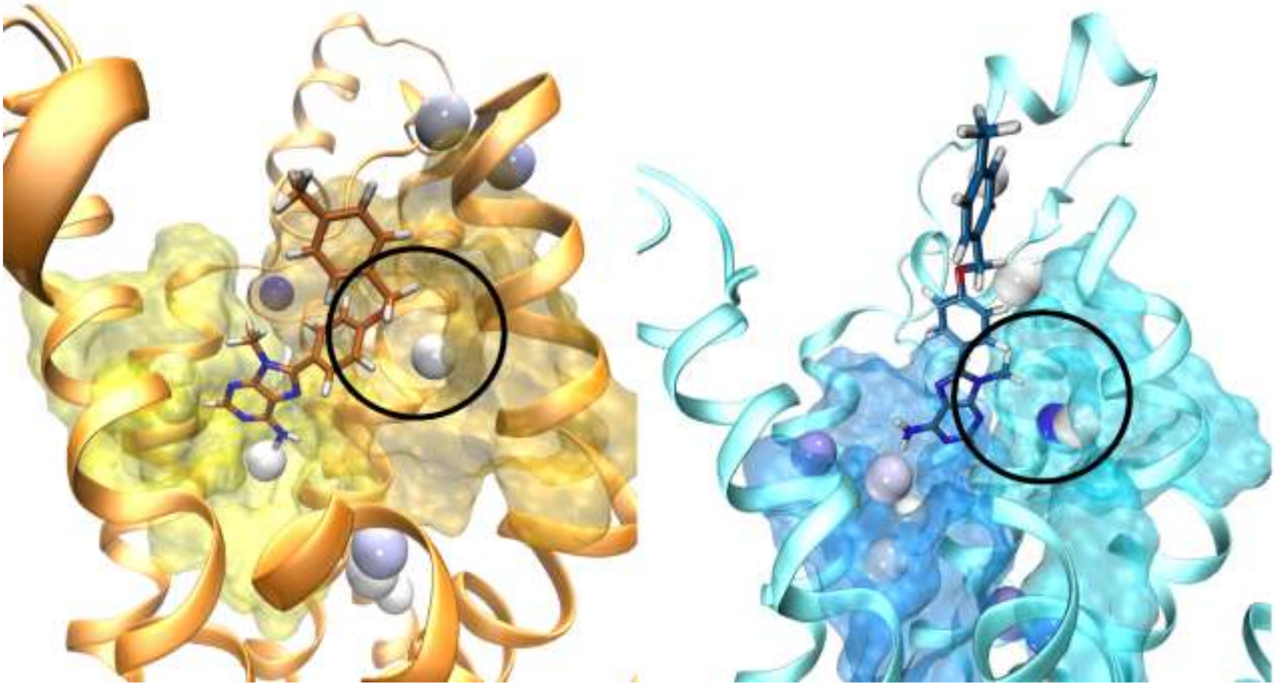

Figure S17 LC4 within the binding site of A_1-AR (on the left) and of on A_{2A}-AR (on the right). In the first case the ligand interacts with the stationary water molecule in the hydrophobic pocket with an oxygen atom, while in the latter case with a methyl group.
